# Reliability and Validity of Shore Hardness in Plantar Soft Tissue Biomechanics

**DOI:** 10.3390/s24020539

**Published:** 2024-01-15

**Authors:** Redent Tonna, Panagiotis E. Chatzistergos, Otis Wyatt, Nachiappan Chockalingam

**Affiliations:** 1 Department of Engineering, School of Digital, Technologies and Arts, Staffordshire University, Stoke-on-Trent ST4 2DE, UK; redent.tonna@staffs.ac.uk; 2 Centre for Biomechanics and Rehabilitation Technologies, Staffordshire University, Stoke-on-Trent ST4 2DE, UK; otis.wyatt@staffs.ac.uk (O.W.); n.chockalingam@staffs.ac.uk (N.C.)

**Keywords:** mechanical tests, hardness tests, elasticity imaging techniques, shear wave elastography, reliability and validity, soft tissue injuries, foot, diabetic foot

## Abstract

Shore hardness (SH) is a cost-effective and easy-to-use method to assess soft tissue biomechanics. Its use for the plantar soft tissue could enhance the clinical management of conditions such as diabetic foot complications, but its validity and reliability remain unclear. Twenty healthy adults were recruited for this study. Validity and reliability were assessed across six different plantar sites. The validity was assessed against shear wave (SW) elastography (the gold standard). SH was measured by two examiners to assess inter-rater reliability. Testing was repeated following a test/retest study design to assess intra-rater reliability. SH was significantly correlated with SW speed measured in the skin or in the microchamber layer of the first metatarsal head (MetHead), third MetHead and rearfoot. Intraclass correlation coefficients and Bland–Altman plots of limits of agreement indicated satisfactory levels of reliability for these sites. No significant correlation between SH and SW elastography was found for the hallux, 5th MetHead or midfoot. Reliability for these sites was also compromised. SH is a valid and reliable measurement for plantar soft tissue biomechanics in the first MetHead, the third MetHead and the rearfoot. Our results do not support the use of SH for the hallux, 5th MetHead or midfoot.

## 1. Introduction

The main role of the soft tissues of the sole of the foot (also known as plantar soft tissue) is to act as a shock absorber to dampen the effect of ground reaction forces during weight-bearing activities by promoting a more even distribution of plantar loads [1,2]. Previous research has demonstrated that specific changes in the mechanical behaviour of plantar soft tissue can significantly undermine the tissue’s ability to fulfil its mechanical role, making it more vulnerable to overloading and injury [3]. The potential for increased risk for an overload injury due to altered tissue biomechanics is particularly important in the case of diabetic foot complications [4,5,6,7,8].

People with diabetes, over time, tend to lose the protective sensation of pressure and pain in their feet due to peripheral neuropathy. As a result, they can repeatedly overload their feet without noticing it, leading to the development of open wounds (ulcers) that heal poorly, are prone to infection and can even lead to amputation. In the UK alone, 169 people have a toe, foot or limb amputated due to diabetes every week [9].

Being able to quantify the stiffness of plantar soft tissues as part of everyday clinical practice could enhance the prediction of diabetic foot ulceration [4] and improve its clinical management [5,10,11]. Existing research methods for the quantitative assessment of the mechanical properties of plantar soft tissue are based on the use of complex bespoke testing devices and computational methods which are not applicable for clinical use [2,3,7]. At the same time, the use of clinical methods that are able to map tissue stiffness, such as ultrasound elastography, is currently limited due to their significant cost, the need for specialist training and limited availability in clinics.

Among the relevant clinical elastography methods, shear wave (SW) ultrasound elastography appears to be the gold standard for the quantitative assessment of the biomechanics of soft tissues [6,12,13,14,15,16,17]. SW elastography involves generating SWs inside the imaged tissue and then measuring their propagation speed (also known as SW speed) as these waves expand within the field of view of the ultrasound sensor. In linearly elastic, homogenous and isotropic materials, SW speed can be used to calculate the Young’s modulus of the imaged material [18]. However, the aforementioned assumptions are not met for biological tissues, making the assessment of tissue stiffness challenging [19]. Indeed, the relevant literature has demonstrated that SW speed is correlated with tissue stiffness but it cannot offer a direct assessment of its absolute value [12,14]. Despite this limitation, SW elastography can detect and assess clinically relevant differences between people or changes over time in tissue stiffness [12,14,20,21]. This unique capability combined with the inherent portability and non-invasive nature of ultrasound has made SW ultrasound elastography a very useful tool in conditions associated with biomechanical changes in the affected tissues such as cancer, liver fibrosis, etc. [22,23,24].

SW elastography has been successfully applied [13,25,26,27,28,29] and validated [12] to study plantar soft tissue biomechanics. However, its increased cost and limited availability remain key barriers to wider adoption outside of research. To further explore and fully understand the role of plantar soft tissue biomechanics in diabetic foot ulceration, there is a need for simpler, cost-effective and reliable methods to assess and monitor changes in the mechanical characteristics of the plantar soft tissue. Considering existing screening tools for diabetes complications [5] and the fact that preventative care for diabetic foot complications is mainly offered as part of community care [30], such methods will also have to be applicable outside specialised acute-care clinics to have a meaningful positive impact on diabetic foot outcomes.

The measurement of Shore hardness (SH), using a handheld durometer, has been extensively used to assess soft tissue biomechanics in vivo and appears to be a good candidate to fill this gap [5,10,31,32,33,34,35,36,37]. SH is a measurement of a material’s resistance to indentation and is given a dimensionless value between 0 and 100 with a high value of SH indicating high resistance to indentation. Even though resistance to indentation is related to material stiffness, the interpretation of SH is made challenging by the complex internal structure and non-linear mechanical behaviour of soft tissues.

Aiming to promote better understanding and more effective use of SH in plantar soft tissue biomechanics, a recent numerical analysis indicated that SH is very sensitive to skin thickness and quantifies the macroscopic deformability of bulk tissue [38]. As a result, a measured difference in SH could be either due to a difference in skin thickness or stiffness, due to a difference in subcutaneous tissue stiffness or due to a combination of all of the above [38]. Considering this limitation, it is unclear whether SH remains a valid measure of plantar soft tissue biomechanics capable of detecting clinically relevant differences and changes in tissue stiffness. At the same time, even though SH has been used for some time now, its reliability when used for plantar soft tissue has not been assessed.

This study experimentally assesses the validity and reliability (intra-/inter-rater) of SH for the study of plantar soft tissue biomechanics. If SH is indeed capable of offering a clinically relevant assessment of plantar soft tissue biomechanics, then it should be strongly correlated with established gold standard measures such as SW elastography [12]. Validity and reliability are separately assessed across different plantar sites to account for the complex structure of the foot and differences between rearfoot midfoot and forefoot.

## 2. Materials and Methods

### 2.1. Participants

A sample of convenience of 20 healthy adults was recruited for this study (11 male and 9 female). Their average (±standard deviation) age, body mass and height were 37 y (±13 y), 81 kg (±18 kg) and 1.69 m (±0.07 m), respectively. The specific inclusion criteria were age ≥ 18 and ability to walk unaided. People with a history of structural surgery in the foot (e.g., amputation or bone fusion) or conditions that can affect their sensitivity to pressure (e.g., diabetes) or can cause pain in the feet (e.g., gout or osteoarthritis) were excluded. People who were not able to comfortably lie prone for prolonged periods of time (up to 30′) were also excluded (e.g., due to pregnancy or lower back pain). Ethical approval was granted by Staffordshire University’s ethics committee (SU22-009). All participants provided written informed consent before any data collection took place.

### 2.2. Biomechanical Measurements

#### 2.2.1. Shore Hardness

**Figure 1:**
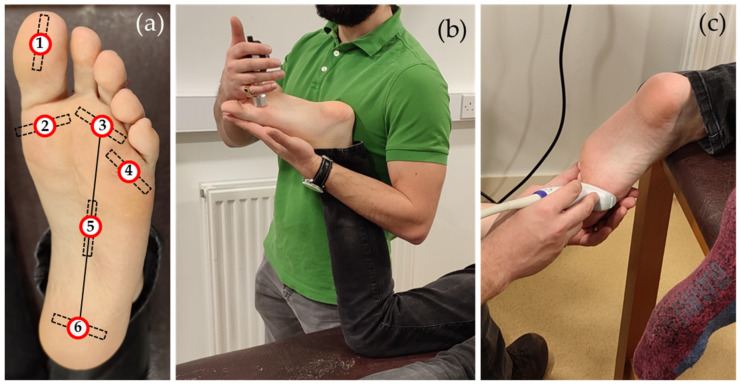
(**a**) The measurement sites and the orientation of the ultrasound probe during SW imaging at the hallux (1), 1st metatarsal head (MetHead) (2), 3rd MetHead (3), 5th MetHead (4), midfoot (5) and rearfoot (6). The testing set-up of SH testing (**b**) and SW elastography imaging (**c**).

SH was measured in different sites of the left foot (Figure 1a) using a Shore-00 durometer (AD-100, Checkline Europe B.V, Dennenweg, The Netherlands). During hardness testing, the participants were asked to lie prone on an examination couch with their shank supported by the examiner at ≈90 degrees to the thigh (Figure 1b). With the left foot relaxed, the durometer was lowered onto each of the plantar measurement sites allowing the tissue to be compressed by the full weight of the device before taking the hardness reading. Each site was tested three times, and their average value was used as the SH measure for each site.

More specifically, measurements were taken at six clinically relevant plantar sites previously used in the literature [5,10,31,32,33,36,37]: the hallux, 1st metatarsal head (MetHead), 3rd MetHead, 5th MetHead, midfoot and rearfoot (Figure 1a). In the cases of the hallux and rearfoot, the examiners were instructed to target the centre of the pulp of the hallux and the centre of the heel, respectively. The three MetHead locations were identified by palpating the apex of the respective MetHeads while the midfoot testing site was defined as the midpoint of an assumed straight line connecting the centre of the heel to the 3rd MetHead (Figure 1a).

To assess inter-rater and intra-rater reliability, SH was measured by two examiners on two different days following a test–retest study design. The retest took place after a minimum of two days and a maximum of 14 days after baseline. To reduce the risk of bias, the testing order was randomised between examiners and special attention was paid to ensure that they were blinded to each other’s results. To improve consistency, testing for each examiner was preceded by preconditioning which involved walking barefoot at a self-selected speed for ≈80 steps [6]. This preconditioning protocol has been demonstrated in the literature to minimise the risk of confounding results due to differences in loading history prior to testing [6].

#### 2.2.2. SW Elastography

Baseline tissue biomechanics was assessed using SW elastography (Supersonic Imaging, Aix-en-Provence, France) and a linear array 4–15 MHz transducer (SuperLinear™ SL15-4). During imaging, individual measurement sites were identified following the same procedure as SH testing (Figure 1). The ultrasound probe was then moved to bring the targeted site to the centre of the imaging window. The probe was kept in place for ≈10 s to ensure that the elastogram had stabilised before capturing an SW elastography image. During ultrasound imaging, the examiner was blinded to the SW elastography results. Considering the effect of pressure on SW speed, special care was given to ensure that a thin layer of gel was always visible between the probe and plantar soft tissue, thus ensuring imaging was consistently performed under minimal compression [12]. The entire imaging process was repeated three times, leading to the recording of a total of three images per site.

**Figure 2:**
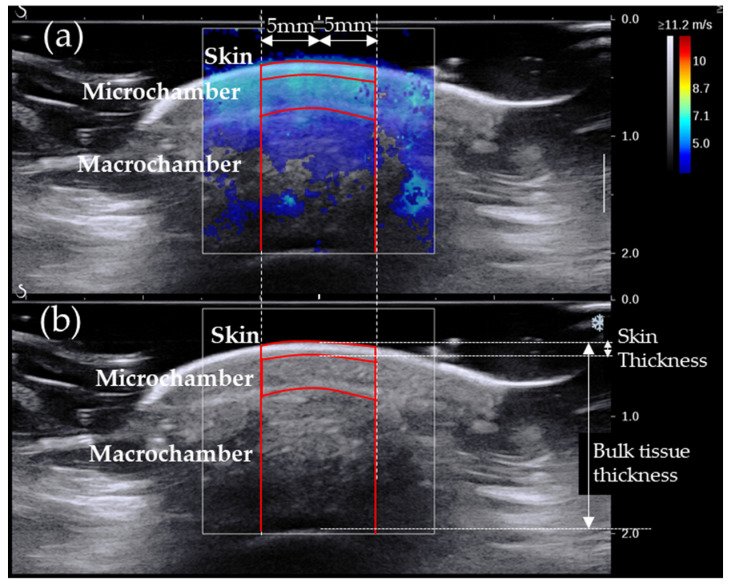
Typical B-mode ultrasound image (**a**) and SW elastogram (**b**) at the heel. The thickness measurements (skin and bulk tissue thickness) and regions where SW speed was measured are also shown (skin, microchamber layer and macrochamber layer).

The recorded ultrasound images were analysed after the end of data collection to measure the thickness and SW speed in different layers of the plantar soft tissue (Figure 2). Bulk tissue thickness (i.e., probe-to-bone distance) and skin thickness were measured at the centre of the image. The SW speed was separately measured for the skin and the microchamber and macrochamber layers [2,8,39]. In each image, the SW speed for individual layers was averaged in an area extending 5 mm on either side of the centre of the image (Figure 2). Measurements were averaged over the available recordings to produce the final results for individual sites.

#### 2.2.3. Statistical Analysis and Sample Size

Spearman correlation analysis was used to assess the association between SH measurements and SW elastography or tissue thickness. For this correlation analysis, SH was averaged between examiners and between test–retest to produce a single representative value per participant for each site. A related-samples Friedman’s two-way analysis of variance by ranks with Bonferroni correction for multiple comparisons was used to assess the significance of differences between regions (a = 0.05).

Intraclass correlation coefficients (ICCs) and Bland–Altman’s limits of agreement (LoA) and bias [40] were calculated for the SH measurements that were recorded: (a) by the two examiners for the same participant during baseline testing (inter-rater reliability) and (b) by the same examiner during two different testing sessions (intra-rater reliability). The ICC values (absolute agreement, two-way mixed, average measures) were interpreted as follows: ICC < 0.40 =  poor, 0.40 ≤ ICC < 0.60 = fair, 0.60 ≤ ICC < 0.74 = good and ICC ≥ 0.75 = excellent reliability [41].

The sample size was calculated to determine whether SH is adequately reliable for research and clinical use. According to established methods for the design of reliability studies, this calculation requires preliminary assumptions about the expected and minimum acceptable values of ICC [42]. Because the present study is the first to assess SH reliability for plantar soft tissue, the assumed ICC value was decided based on the relevant literature on the reliability of SW elastography (expected ICC ≈ 0.90) [26]. The minimum acceptable ICC was decided considering that the reliability of SH should be at least “good” to enable use in research and clinical applications (minimum acceptable ICC = 0.60) [41]. Based on these factors, it was calculated that, for two examiners, an accurate assessment of reliability requires at least eleven participants (α = 0.05; β = 0.20) [42]. Setting a recruitment target of twenty people ensures the robustness of the study even in the case of reasonable worst scenarios regarding missing data and participants being lost to follow-up.

## 3. Results

The planned SH measurements were successfully completed for all 20 participants. SW speed and thickness were also successfully measured for almost all participants except for bulk tissue thickness for the midfoot or rearfoot. Missing data were due to the inability to clearly see the boundary of the underlying bones in the recorded ultrasound images which made it very difficult to confidently measure probe-to-bone distance. As a result, bulk tissue thickness for these two sites was completely excluded from any further analysis.

Representative SH at the rearfoot was statistically significantly greater than the midfoot and all MetHead regions (Table 1). The hallux also appeared to have significantly higher SH than the midfoot and the MetHead regions. SW elastography revealed a consistent pattern for the rearfoot, with it having a significantly higher SW speed than all remaining regions (Table 1). Differences were statistically significant for SW speed measured in the skin, in the microchamber layer and in the macrochamber layer (Table 1). Regarding thickness, statistically significant differences were found only for skin (Table 1). More specifically, the skin at the rearfoot and 3rd MetHead regions was significantly thicker than the midfoot or hallux.

Spearman correlation analysis revealed statistically significant positive associations between SH and skin or microchamber SW speed in the 1st MetHead, 3rd MetHead and rearfoot (Table 2). These correlations were strong for the 3rd MetHead and of moderate strength for the rearfoot [43]. In the case of the 1st MetHead they were strong for the skin and moderate for the microchamber layer. There was no statistically significant correlation between SH and SW speed in the hallux, 5th MetHead or midfoot. In the case of thickness measurements, skin thickness at the 1st MetHead was statistically significantly correlated with SH. This correlation was positive and of moderate strength (r = 0.575, *p* = 0.008, N = 20) [43].

Inter-rater ICC was good or excellent for the 1st MetHead, 3rd MetHead, midfoot and rearfoot (Table 3). It was fair or poor for the hallux and the 5th MetHead, respectively. Intra-rater ICC was good or excellent for both examiners across all tested sites (Table 3).

**Figure 3:**
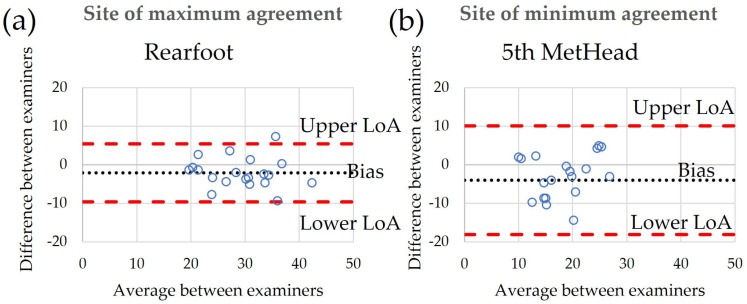
Bland-Altman plots of agreement between examiners during baseline testing for the plantar site with the best (**a**) and worst agreement (**b**) as indicated by their respective bias and limits of agreement (LoA).

**Figure 4:**
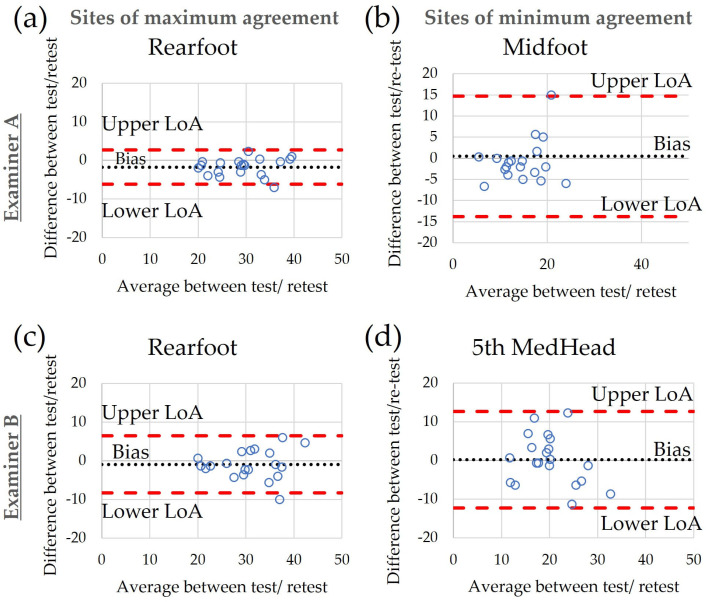
Bland–Altman plots of agreement between test/retest for examiner A (**a**,**b**) and examiner B (**c**,**d**) for the plantar sites with the best (**a**,**c**) and worst agreement (**b**,**d**).

Bland–Altman plots of inter-examiner reliability indicated that bias remained relatively low, ranging from −4.0 to 4.2 (Table 3). LoA were narrowest for the rearfoot and widest for the 5th MetHead, indicating maximum and minimum agreement between examiners, respectively (Figure 3). Bland–Altman plots of intra-examiner reliability showed bias ranging between −3.0 and 0.5 (Table 3). Similar to before, LoA were narrowest for the rearfoot for both examiners, indicating maximum levels of agreement between test–retest. For examiner A, they were widest for the midfoot while for examiner B, they were widest for the 5th MetHead, indicating the lowest levels of agreement for these two sites (Figure 4). A detailed table of bias and LoA for all tested regions can be found in Appendix A (Table A1).

## 4. Discussion

This study presents the first assessment of the validity and reliability of SH for plantar soft tissue. The validity was tested by comparing SH with SW elastography, namely the gold standard clinical method to study soft tissue biomechanics. Statistically significant correlations between these two methods were found for the first MetHead, the third MetHead and rearfoot, directly linking increased plantar soft tissue stiffness with increased SH. No statistically significant association was found for the hallux, the 5th MetHead or for the midfoot. These results support the validity of SH for the study of plantar soft tissue biomechanics in the first MetHead, third MetHead and rearfoot.

Statistically significant associations between SH and SW speed were observed for the skin and the microchamber layer. Moreover, increased SH was also significantly correlated with increased skin thickness. These observations validate a previous finite element study indicating that SH is equally affected by the thickness of skin, the stiffness of skin and the stiffness of subcutaneous tissue [38]. As a result, it can be concluded that SH should be interpreted as a measure of the macroscopic deformability of bulk tissue biomechanics. Moreover, it should be complemented by measurements of skin thickness to enable drawing conclusions about tissue differences or changes in tissue stiffness. Even though the present study focused on the plantar soft tissue, these conclusions can be generalised to inform the correct use of SH across different anatomical areas.

The ICC results and Bland-Altman’s LoA indicated that SH has satisfactory reliability (intra-/inter-rater) when used for the first MetHead, the third MetHead and the rearfoot. However, its reliability appears to be compromised for the hallux, 5th MetHead and midfoot. Unsatisfactory reliability in these sites could be caused by inconsistencies in durometer placement due to the nature of the morphology of these areas.

Bringing together observations about validity and reliability enables us to conclude that SH is a useful and relevant measure of plantar soft tissue biomechanics for specific areas of the foot. More specifically, our results support its use for the first MetHead, the third MetHead and the rearfoot but not for the hallux, the 5th MetHead or the midfoot. At this point, it is difficult to explain why SH appears to be reliable and valid for some sites and not for others. Site-specific protocols and adaptations of conventional SH could be considered in future research to address this apparent limitation of SH testing.

This investigation was limited to six specific plantar sites that have been previously studied in the literature using SH and which coincide with areas where diabetic foot ulceration is likely to develop [5,31,32,33,36,37]. Being limited to only six sites, this study cannot conclude on the full extent of the areas where SH is valid and reliable but demonstrates that this is unlikely to be the entire plantar surface.

This observation can inform better use of SH in research by focusing data collection in areas where its validity and reliability are established (first MetHead, third MetHead and rearfoot). Having said that, it is important to highlight that SH remains a valid and reliable measure when used within its limitations [5,10,33,36].

Accounting for this newly observed limitation of SH is particularly important for the exploration and development of future clinical applications. More specifically, the inability to map plantar soft tissue biomechanics across the surface of the sole of the foot means that SH (most likely) is unsuitable for applications where the detection of localised differences or changes in plantar soft tissue biomechanics is important.

According to the current literature, measurements of plantar soft tissue biomechanics could enhance the clinical management of diabetic foot ulceration: (a) by enhancing risk assessment and patient stratification [4], (b) by enabling the indirect assessment of plantar loading [10] and (c) by enabling the direct detection of overload injuries before they become visibly obvious [6]. Further research is needed to explore whether SH remains a good candidate method for the aforementioned specific applications.

This study was limited to younger healthy volunteers, who are not the typical population SH would be used on in clinical practice. Including older people and people with diabetes would affect the median values and range of SH and SW speed. However, these potential differences in the absolute values of results do not affect the generalizability of findings regarding the validity and reliability of SH.

## 5. Conclusions

When SH is used in vivo to study layered tissues, such as skin and subcutaneous tissue, it should be interpreted as a measure of the macroscopic capacity of the bulk tissue to deform (i.e., skin and subcutaneous tissue combined). SH should be accompanied by measurements of skin thickness to enable translating this assessment of macroscopic deformability into an assessment of bulk tissue stiffness. When used for the plantar soft tissue, SH appears to be a valid and reliable measurement for plantar soft tissue biomechanics in the first MetHead, the third MetHead and the rearfoot. Our results do not support the use of SH for the hallux, 5th MetHead or the midfoot.

## Figures and Tables

**Table 1:** Median (min. and max. values) of biomechanical measurements for different plantar foot regions and layers.

	SH	SW Speed (m/s)			Thickness (mm)	
		*Skin*	*Micro-* *Chamber*	*Macro-* *Chamber*	*Skin*	*Bulk* *Tissue*
**Hallux**	26 (13, 40)	4.3 (2.8, 5.3)	3.7 (2.5, 4.8)	2.5 (1.7, 4.3)	0.9 (0.6, 1.0)	11.5 (9.0, 14.4)
**1st MetHead**	18 (4, 26)	4.1 (2.8, 7.0)	3.4 (2.5, 5.0)	2.0 (1.5, 3.3)	0.9 (0.7, 3.2)	13 (11.2, 15.5)
**3rd MetHead**	15 (5, 28)	4.3 (3.1, 6.4)	3.7 (2.7, 5.1)	1.9 (1.3, 3.0)	1.1 (0.9, 3.6)	12.6 (10.3, 14.5)
**5th MetHead**	16 (10, 28)	4.6 (3.2, 5.8)	3.7 (2.7, 5.2)	2.3 (1.6, 4.2)	1.0 (0.6, 1.3)	12.3 (10.8, 13.9)
**Midfoot**	16 (6, 27)	3.2 (2.6, 4.6)	3.0 (2.4, 4.5)	2.3 (1.6, 3.6)	0.8 (0.6, 1.2)	-
**Rearfoot**	30 (20, 41)	6.6 (3.7, 10.1)	5.5 (3.6, 7.7)	3.1 (1.6, 5.1)	1.1 (0.8, 2.1)	-

**Table 2:** Statistically significant correlations between SH and SW speed.

		r	*p* (2-Tailed)	N
**1st MetHead**	Skin	0.715	<0.001	20
	Microchamber	0.634	0.003	20
**3rd MetHead**	Skin	0.865	<0.001	20
	Microchamber	0.894	<0.001	20
**Rearfoot**	Skin	0.638	0.004	18
	Microchamber	0.659	0.003	18

r: correlation coefficient, *p*: level of significance (2-tailed), N: sample size. Moderate correlation strength: 0.4 < r ≤ 0.7, strong: 0.7 < r ≤ 0.9 and very strong: 0.9 < r ≤ 1.0 [43].

**Table 3:** Inter-/intra-rater reliability assessed by the respective ICC scores.

Inter-Rater	ICC	95% CI		
Hallux	0.592	0.158, 0.824		
1st MetHead	0.623 *	0.271, 0.830		
3rd MetHead	0.636 *	0.283, 0.838		
5th MetHead	0.295	−0.090, 0.627		
Midfoot	0.616 *	0.262, 0.826		
Rearfoot	0.797 **	0.515, 0.918		
**Intra-rater**	**Examiner A**		**Examiner B**	
	ICC	95% CI	ICC	95% CI
Hallux	0.862 **	0.638, 0.947	0.653 *	0.299, 0.848
1st MetHead	0.953 **	0.886, 0.981	0.762 **	0.477, 0.900
3rd MetHead	0.852 **	0.664, 0.939	0.744 *	0.455, 0.891
5th MetHead	0.870 **	0.706, 0.946	0.620 *	0.251, 0.831
Midfoot	0.655 *	0.309, 0.848	0.729 *	0.441, 0.882
Rearfoot	0.902 **	0.620, 0.967	0.838 **	0.641, 0.932

ICC: interclass correlation coefficient, CI: confidence intervals. *: good/**: excellent reliability [41].

## Data Availability

The data that support the findings of this study are available from the corresponding author upon reasonable request.

## Appendix A

**Table A1:** Bland–Altman bias and limits of agreement (LoA) for all tested sites.

Inter-Rater Agreement	Bias	Upper LoA	Lower LoA
Hallux	4.2	16.5	−8.1
1st MetHead	1.4	11.5	−8.8
3rd MetHead	−2.8	9.5	−15.2
5th MetHead	−4.0	10.1	−18.1
Midfoot	−1.6	9.1	−12.2
Rearfoot	−2.1	5.5	−9.6
**Intra-rater agreement:** Examiner A			
Hallux	−2.1	5.3	−9.4
1st MetHead	−0.1	3.6	−3.8
3rd MetHead	−0.5	8.6	−9.5
5th MetHead	−0.1	8.9	−9.1
Midfoot	0.5	14.8	−13.8
Rearfoot	−1.7	2.7	−6.2
**Intra-rater agreement:** Examiner B			
Hallux	−3.0	8.8	−14.7
1st MetHead	−2.0	6.6	−10.7
3rd MetHead	0.3	10.8	−10.3
5th MetHead	0.2	12.7	−12.3
Midfoot	−0.9	9.6	−11.3
Rearfoot	−1.0	6.4	−8.3
